# Evaluation of the Clinical Effectiveness of the Salmeterol/Fluticasone Fixed-Dose Combination Delivered via the Elpenhaler^®^ Device in Greek Patients with Chronic Obstructive Pulmonary Disease and Comorbidities: The AEOLOS Study

**DOI:** 10.3390/jpm11111159

**Published:** 2021-11-08

**Authors:** Paschalis Steiropoulos, Stavros Tryfon, Christos Kyriakopoulos, Konstantinos Bartziokas, Konstantinos Kostikas

**Affiliations:** 1 Respiratory Medicine Department, Faculty of Medicine, Democritus University of Thrace, 68131 Alexandroupolis, Greece; pstirop@med.duth.gr; 2 Respiratory Medicine Department, “G. Papanikolaou” General Hospital of Thessaloniki, 57010 Thessaloniki, Greece; stavrostryfon@yahoo.gr; 3 Respiratory Medicine Department, Faculty of Medicine, University of Ioannina, 45500 Ioannina, Greece; ckyriako123@gmail.com (C.K.); bartziokas@gmail.com (K.B.)

**Keywords:** COPD, FEV_1_, mMRC, salmeterol and fluticasone propionate fixed-dose combination, safety analysis

## Abstract

Background: Chronic Obstructive Pulmonary Disease (COPD) is an inflammatory lung disease characterized by airflow limitation that is not completely reversible. The fixed-dose combination of salmeterol and fluticasone propionate (SFC) has been approved as a treatment for COPD patients with a history of recurrent exacerbations and significant symptoms despite regular bronchodilator therapy. In the present study, we evaluated the change in FEV_1_, mMRC dyspnea score and satisfaction in COPD patients with at least one comorbidity versus those without comorbidities treated with a fixed-dose SFC via the Elpenhaler^®^ device for 12 months. Methods: A 12-month multicenter prospective, observational study (NCT02978703) was designed. Data were collected during the enrollment visit (V0) and six (V1) and twelve months (V2) after the initiation of treatment with Elpenhaler^®^ SFC. The evaluation of the efficacy of the fixed-dose SFC was performed by assessing the change in lung function and dyspnea as expressed by FEV_1_ and the mMRC dyspnea scale score in COPD patients with and without comorbidities. Results: In total 1016 patients were enrolled, following usual daily clinical practice. A statistically significant improvement was observed in FEV_1_ in the total study population between visits V0, V1 and V2, with a change from the baseline at V1 0.15 ± 0.22 L and at V2 0.21 ± 0.25 L (*p* < 0.0001 for both comparisons). This improvement was exhibited regardless of the COPD severity at the baseline, being more noticeable in GOLD 2020 groups B and C. Similarly, a significant improvement was observed in mMRC dyspnea scale values between successive visits (*p* < 0.0001). In patients without comorbidities, there was a significant improvement in FEV_1_ of 0.19 ± 0.24 L at V1 and 0.28 ± 0.27 L at V2 (*p* < 0.0001 for both comparisons), as well as in the mMRC dyspnea score (*p* < 0.0001). In patients with at least one comorbidity, a corresponding but smaller improvement in FEV_1_ was observed (0.11 ± 0.34 L at V1 and 0.20 ± 0.42 L at V2; *p* < 0.0001 for both comparisons and in the mMRC score (*p* < 0.0001). In the multiple linear regression analysis BMI, GOLD 2020 groups, mMRC and the presence of comorbidities at the baseline were significant factors for the change of FEV_1_ between V0 and V2. Conclusions: COPD patients treated for twelve months with SFC via the Elpenhaler^®^ device showed significant improvement in lung function and dyspnea at 6 and 12 months, irrespective of the presence of comorbidities.

## 1. Introduction

Chronic obstructive pulmonary disease (COPD) is an inflammatory chronic disease of the airways characterized by persistent respiratory symptoms and airflow limitation. Despite the fact that it is preventable and treatable, it remains a major public health challenge that is mostly caused by continuous exposure to risk factors (mainly tobacco but also biomass fuels) as well as in the aging population [1,2]. It is currently the third leading cause of death, responsible for approximately 6% of the world’s total deaths (approximately 3.3 million annually) [3] and is the 7th leading cause of disability adjusted life years (DALYs) worldwide [1]. COPD patients from countries with lower socioeconomic statuses have been reported to have worse outcomes than patients from countries with higher statuses [4]. This may be due to the difficulty that patients in these countries face in accessing health-care facilities, including affordable inhaled COPD therapies [5,6].

Current drugs can reduce COPD symptoms and the frequency of exacerbations and improve health status and exercise capacity [2]. Inhaled bronchodilators are the cornerstone to the management of COPD symptoms. Recent studies have shown that combination therapy of long-acting β_2_-agonists and inhaled corticosteroids (LABA/ICS) has greater efficacy than LABA alone in improving lung function, health status and exacerbation frequency [2,7]. There are currently several LABA/ICS combinations on the Global market that differ in the pharmacokinetics and dosage of the two active ingredients they contain [8]. Salmeterol xinafoate/fluticasone propionate combination (SFC) therapy is a LABA/ICS that has been widely studied in COPD, showing significant improvements in lung function, exacerbation frequency and health status compared to mono-bronchodilators in randomized controlled trials [8,9].

The stringent patient selection in randomized controlled trials (RCTs) makes them less representative of the real-life COPD patient population, thus the use of nationwide databases to conduct real-life studies contributes to examining longer term outcomes, providing information to complement the results of RCTs. Observational studies allow the assessment of patients normally excluded from RCTs, such as those with comorbidities that are often excluded from RCTs. Real-world observational studies cast a wider investigation net through the consideration of unselected, representative patients managed in real-life clinical practice [10,11].

The primary objective of the current real-life study was to evaluate changes in lung function in patients with COPD receiving the fixed-dose SFC via the Elpenhaler^®^ device for 12 months. Secondary objectives were the evaluation of dyspnea, patient satisfaction from Elpenhaler^®^ device after 6 months of treatment and adverse events (AΕs).

## 2. Materials and Methods

### 2.1. Patients

**Figure 1:**
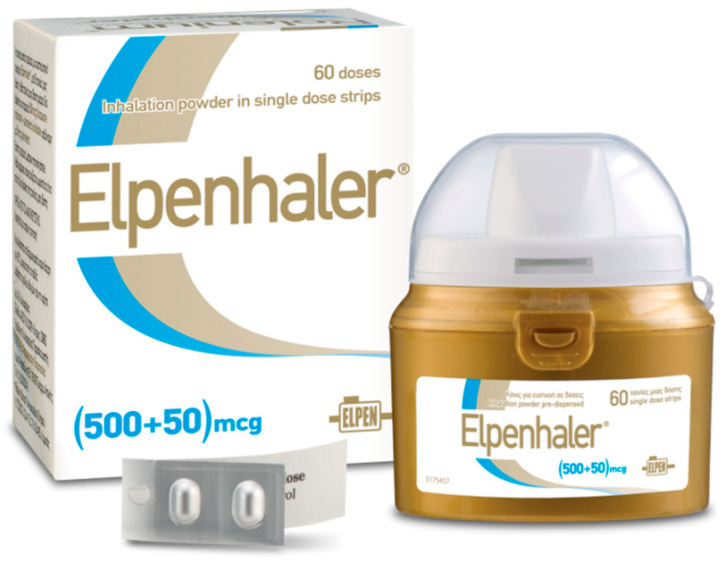
Elpenhaler^®^ device with the fixed-dose combination of salmeterol/fluticasone propionate (SFC) 50/500 μg.

We enrolled consecutive patients with an established diagnosis of COPD, by 71 institutions and private practices in different regions in Greece, between May 2017 and December 2017. All subjects were adults (>18 years) with an established diagnosis of COPD by a pulmonologist, a FEV_1_ < 60% (predicted) and a history of exacerbations who were symptomatic despite usual treatment with bronchodilators. All study participants were under newly initiated treatment with a fixed-dose combination of salmeterol/fluticasone propionate (SFC), 50/500 μg through inhalation via the Elpenhaler^®^ device (Figure 1). Patients with inability or unwillingness to cooperate with the investigators or without available spirometry data were excluded. We also excluded patients who used or had recently used treatment with ICS for COPD (during the last 3 months prior to study enrollment). All study participants were invited to participate in the present study on the first day of their evaluation by a study investigator. All patients received the assurance that their care would not be affected by their decision to participate in the study. The study was conducted according to the guidelines of the Declaration of Helsinki and approved by the Institutional Review Board of the twelve hospitals that participated in the study: General Hospital of Patras (IRB 10 May 2017), General Hospital of Athens “Sotiria”(IRB 8 February 2017), General Hospital of Rethymno (IRB 23 February 2017), General Hospital of Chania (IRB 1 March 2017), University General Hospital of Athens “Attikon”(IRB 13 March 2017), Sismanoglio-Amalia Fleming General Hospital of Athens (IRB 30 May 2017), General Hospital of Thessaloniki “G. Gennimatas-Agios Dimitrios”(IRB 21 February 2017), General Hospital of Grevena (IRB 10 March 2017), General Hospital of Kavala (IRB 6 April 20217), General Hospital of Drama (IRB 1 March 2017), General Hospital of Imathia (IRB 17 February 2017), and Papanikolaou General Hospital of Thessaloniki (IRB 26 June 2017). The study protocol was approved by the local ethics committee and all participants provided written informed consent.

### 2.2. Study Design

This was an open-label, 12-month observational, prospective, multicenter study of patients diagnosed with COPD for whom the treating physicians had made a decision to prescribe the combination of salmeterol/fluticasone in the Elpenhaler^®^ device (NCT02978703). Patient socio-demographics were recorded, including age, gender, body mass index (BMI), smoking status, history of exacerbations, current treatment for COPD and comorbidities with an emphasis on cardiovascular disease (arterial hypertension, congestive heart failure, coronary artery disease, arrhythmias or stroke) and metabolic diseases (diabetes mellitus type ΙΙ and dyslipidemia). Clinical parameters were also collected on admission, including vital signs and degree of dyspnea (modified Medical Research Council, mMRC, scale) [12].

The mMRC score is a five-item questionnaire in which patients categorize their own level of disability [12]. Patients are characterized as grade 0 or 1, “Short of breath with strenuous exercise or when hurrying”; grade 2, “Walk slower than people of the same age on the level or stop for breath while walking at own pace on the level”; grade 3, “Stop for breath after 100 yards or after a few minutes on the level” and grade 4, “Too breathless to leave the house”.

The satisfaction of the patients from the use of the Elpenhaler^®^ device was assessed by the Feeling of Satisfaction with the Inhaler (FSI-10) questionnaire [13,14]. The FSI-10 (includes 10 questions) is a self-administered questionnaire which evaluates patient satisfaction with their inhaler device. The answer options vary from “hardly at all” (score of 1 on a 5-point Likert scale) to 5 “very” (score of 5). Consequently, the total score ranges from 10 to 50, with higher scores indicating better satisfaction. The FSI-10 questionnaire also assesses patient opinions regarding ease or difficulty of use, portability and usability of devices for delivery of inhaled drugs. The diagnosis and classification of airflow limitation was based on a post-bronchodilator spirometry in stable condition, according to the GOLD recommendations (patients were classified as GOLD stages 1–4) [15].

The initial evaluation was made during the enrollment visit (V0) and patients were re-evaluated after six (6) months (V1) and after twelve (12) months (V2) from the start of treatment with SFC via the Elpenhaler^®^ device. In those visits, a study investigator recorded the patient’s degree of dyspnoea by using the mMRC scale and the change of pulmonary function parameters by performing spirometry.

### 2.3. Study Outcomes

The primary endpoint was change in usual lung function parameters (FEV1, FVC, FEV1/FVC) at 12 months (±2 weeks) from the baseline, in patients with and without comorbidities, and based on COPD severity (GOLD 2020 groups, GOLD spirometric stages and mMRC dyspnea scale). Secondary outcomes included change in dyspnea (mMRC scale), the presence of comorbidities, patient satisfaction from Elpenhaler after 6 months of treatment as assessed by the FSI-10 questionnaire and adverse events (AΕs). All AEs were summarized according to the Medical Dictionary for Regulatory Activities (MedDRA) and specifically by the system organ class (SOC) and preferred term (PT).

### 2.4. Statistical Analysis

Continuous variables were summarized with the use of descriptive statistical measures (mean value, standard deviation (SD), median and (IQR)). For the mean change in primary and secondary endpoints, 95% CIs were provided. Descriptive statistics were also used in summarizing changes between 0–6 months, 6–12 months and 0–12 months. Categorical variables were displayed as frequency tables (N, %). A chi-squared test was used to identify possible associations between categorical parameters. Analyses were performed for the total study population, as well as for several subgroups (upon data availability) associated with comorbidities (patients with no comorbidities, with at least one comorbidity and with cardiovascular and/or metabolic diseases). The Kolmogorov–Smirnov test of normality was performed before the assessment of a statistical test. In case of non-normality of the data, non-parametric methods were used. All the statistical tests were two-sided and were performed at a 0.05 significance level. The primary endpoint was evaluated by a change in FEV_1_, FVC and FEV_1_/FVC from the baseline at 12 months using a paired *t*-test (or the equivalent non-parametric Wilcoxon signed-rank test). The aforementioned analysis was performed for the total study population as well as for the patients with (a) no comorbidities, (b) at least one comorbidity and (c) with cardiovascular and or metabolic diseases. Changes in all spirometry results from the baseline at 6 and 12 months as well as between 6 and 12 months were assessed by paired *t*-tests or equivalent non-parametric Wilcoxon signed-rank tests. Additionally, changes in all spirometry results between 6 and 12 months were assessed by AN.CO.VA using baseline spirometry values as covariates. Similar analyses (in the total population and in subgroups) were performed (if applicable) for the evaluation of the mMRC dyspnea scale and the FSI-10 questionnaire (6 months only). Moreover, in order to define the determining factors for the change of FEV_1_ between the baseline visit and V2, a univariate and a multiple linear regression analysis were performed where we included demographic characteristics (age, gender and BMI), as well as COPD severity parameters (GOLD 2020 groups, mMRC and FEV_1_ at the baseline) and the presence of comorbidities. AEs were assessed in terms of incidence, severity and causality to the study drug while they were summarized by the System Organ Class (SOC) and the Preferred Term (PT) using the MedDRA dictionary version 22.0.

## 3. Results

### 3.1. Demographic and Other Baseline Characteristics

The current study included 71 sites in Greece, which overall enrolled 1016 patients; 992 patients (97.6%) completed the 6-month follow-up, and 966 patients (95.1%) completed the 12-month follow-up. The majority of the patients were males (74%) with a mean age of 69.5 years (Table 1). Of these patients, 40.5% were current smokers with a mean number of 59-pack years, while 52.8% were ex-smokers (Table S1). The mean duration of COPD from diagnosis up to the study initiation was 7.3 years. The most common symptom reported was dyspnea (31.7%); using the mMRC scale, 76.6% of the patients reported a dyspnea of grade 2 or 3. The majority of the study participants (93.9%) had moderate or severe airflow limitation (46.4% spirometric stage 2 and 47.5% stage 3), and 66.4% of the patients were classified in GOLD 2020 groups C and D (Table 1). Moreover, 688 patients (67.7%) were vaccinated at least once, with 482 patients being vaccinated for both influenza and pneumococcus (Table S2).

Τhe most common treatment schemes previously received by the patients were LABA/LAMA combinations (39.4%) followed by LAMA (29.8%), while 199 patients (19.6%) had not received any COPD treatment previously (Table 1). All participants received SFC Elpenhaler^®^ at study initiation. Of those, 421 patients (41.4%) received only the study drug as monotherapy, while 595 patients (58.6%) received SFC Elpenhaler^®^ in combination with other COPD treatments. The most frequently administered combination therapy during the study was LABA/LAMA/ICS (47.8%), i.e., SFC Elpenhaler^®^ in combination with LAMA (Table S3).

### 3.2. Comorbidities in Study Participants

As shown in Table 2, 238 patients (23.4%) had no comorbidities while the remaining 778 patients (76.6%) had at least one comorbidity. Additionally, 711 (69.9%) patients had at least one cardiovascular and/or metabolic disorder, and 415 patients (40.8%) had only one cardiovascular disorder, while 283 patients (27.9%) had only one metabolic disorder, and 289 patients (28.4%) had >2 comorbidities. Of the 778 patients with at least one comorbidity, 653 (83.9%) had a cardiovascular disease followed by metabolic diseases (373 patients, 47.9%).

### 3.3. Improvement in Lung Function at 6 and 12 Months in Patients with and without Comorbidities

**Figure 2:**
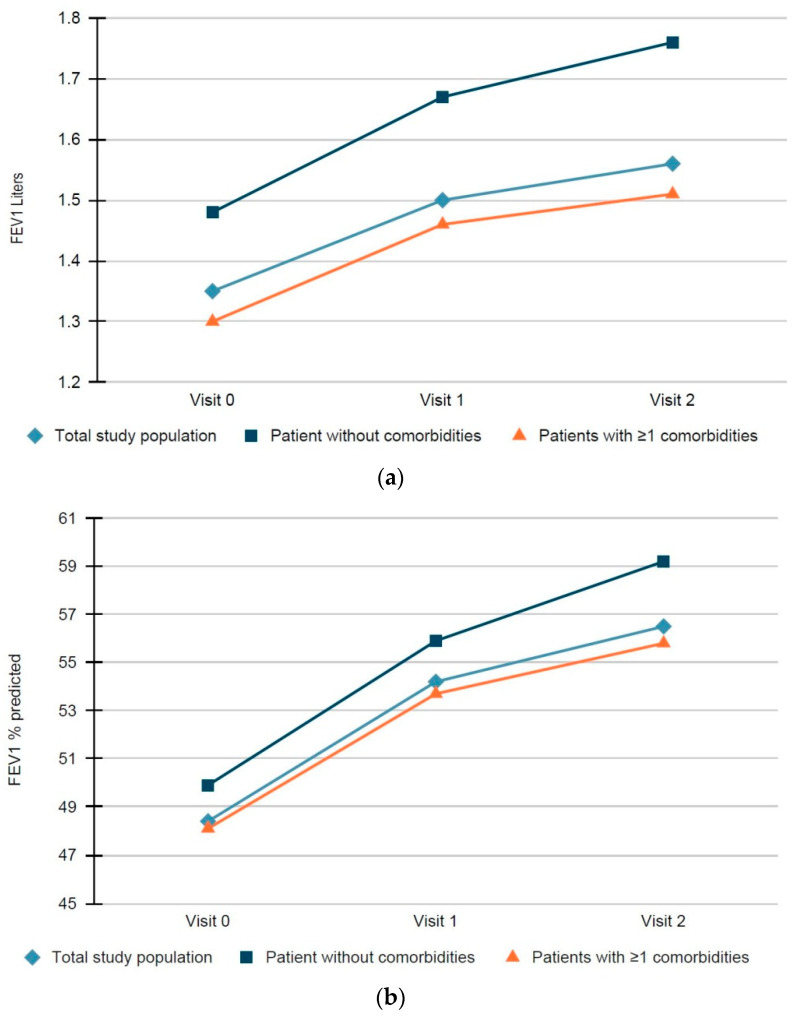
(**a**) FEV_1_ (Liters) per visit in total study population, patients without comorbidities and with ≥1 comorbidities; (**b**) FEV_1_% predicted per visit in total study population, patients without comorbidities and with ≥1 comorbidities.

A statistically significant improvement was observed in FEV_1_ in the total study population between visits V0, V1 and V2. The change from the baseline at V1 was 0.15 ± 0.22 L and at V2 was 0.21 ± 0.25 L (*p* < 0.0001 for both comparisons). Similar changes were observed for the FVC and FEV_1_/FVC ratio. A statistically significant change was also observed in FEV_1_ and FVC between 6 and 12 months (*p* < 0.0001 for both comparisons) (Table 3, Figure 2a,b).

Similarly, in the patients without comorbidities, the change in FEV_1_ from the baseline at V1 was 0.19 ± 0.24 L and at V2 0.28 ± 0.27 L (*p* < 0.0001 for both comparisons). Similar changes were observed for the FVC and FEV_1_/FVC ratio. A statistically significant change was also observed in FEV_1_ and FVC between 6 and 12 months (*p* < 0.0001 and *p* = 0.004, respectively) (Table S4, Figure 2a,b). For the patients with at least one comorbidity, the change in FEV_1_ from the baseline at V1 was 0.11 ± 0.34 L and at V2 0.20 ± 0.42 L (*p* < 0.0001 for both comparisons). Similar changes were observed for the FVC and FEV_1_/FVC ratio. A statistically significant change was also observed in FEV_1_ and FVC between 6 and 12 months (*p* < 0.0001 for both comparisons) (Table S5, Figure 2a,b). The improvement of FEV_1_ from the baseline was more pronounced among patients without comorbidities both at V1 (*p* = 0.017) and at V2 (*p* = 0.034).

In the patients with cardiovascular and/or metabolic comorbidities, FEV_1_ was significantly increased at 6 and 12 months from the baseline. The change from the baseline at V1 was 0.14 ± 0.21 L and at V2 0.20 ± 0.24 L (*p* < 0.0001 for both comparisons). The same applied for the FVC, FEV_1_/FVC ratio. A statistically significant change was also observed in FEV_1_ predicted and FVC between 6 and 12 months (*p* < 0.0001 for both comparisons) (Table S6).

### 3.4. Improvement in Lung Function at 6 and 12 Months According to COPD Severity

**Figure 3:**
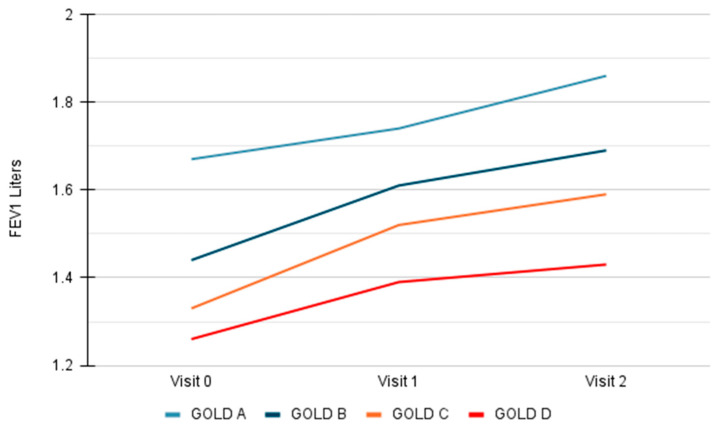
FEV_1_ (Liters) per visit based on GOLD COPD 2020 groups.

In the total study population SFC contributed to the FEV_1_ improvement regardless of the GOLD 2020 group at the baseline, being more profound for groups B (ΔFEV_1_,V0–V1: 0.17 ± 0.19; V0–V2: 0.25 ± 0.24; V1–V2: 0.07 ± 0.15) and C (ΔFEV_1_,V0–V1: 0.19 ± 0.22; V0–V2: 0.26 ± 0.27; V1–V2: 0.07 ± 0.23), reaching statistical significance between visits V0–V1, V0–V2 and V1–V2 (*p* = 0.015, <0.001 and 0.014, respectively) (Table 4, Figure 3).

Improvement in FEV_1_ between visits V0, V1 and V2, was also exhibited in all GOLD spirometric stages, being more noticeable in patients categorized in stage 3 (Table S7). Finally, a numerical improvement was observed in FEV_1_ between visits V0, V1 and V2 according to the MRC dyspnea scale classification, being more obvious in stages 0–2 (Table S8).

In order to define the determining factors for the change of FEV_1_ between the baseline visit and V2, univariate and multiple linear regression analyses were performed. BMI, GOLD 2020 groups, mMRC and the presence of comorbidities at the baseline were significant factors in both the univariate and multiple linear regression analyses for the change of FEV_1_ (Table 5).

### 3.5. Improvement in Dyspnea (mMRC) in Patients with and without Comorbidities

**Figure 4:**
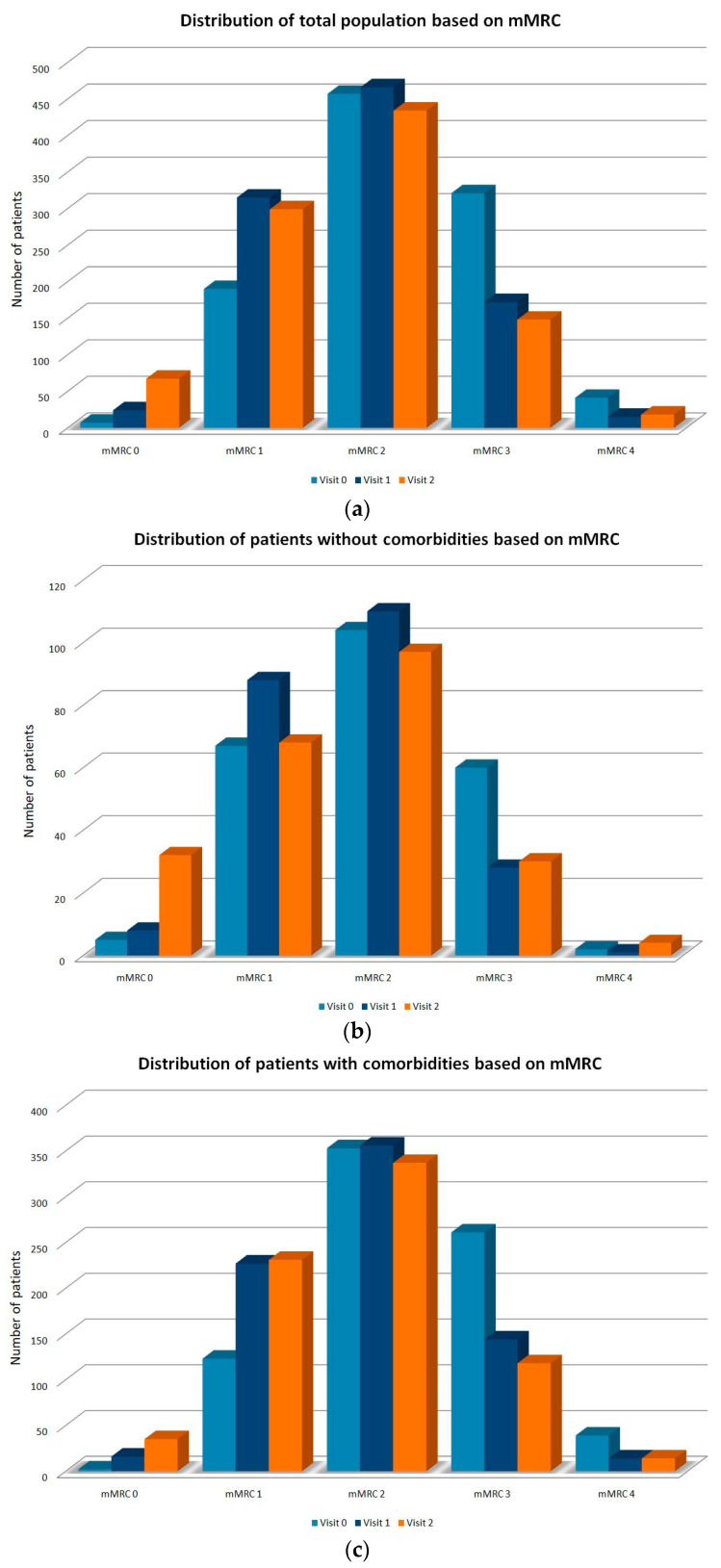

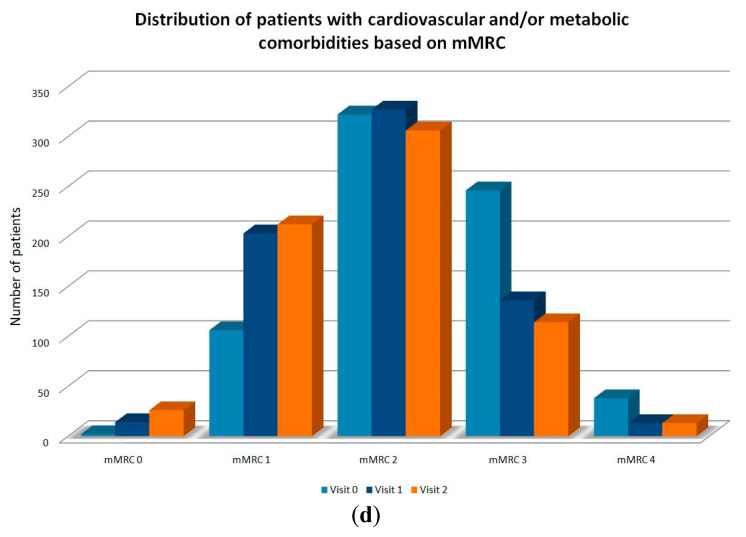
(**a**) Distribution of the total number of patients per visit based on mMRC dyspnea scale; (**b**) Distribution of patients without comorbidities per visit based on mMRC dyspnea scale; (**c**) Distribution of patients with comorbidities per visit based on mMRC dyspnea scale; (**d**) Distribution of patients with cardiovascular and/or metabolic comorbidities per visit based on mMRC dyspnea scale.

The majority of the patients in the total study population had an mMRC dyspnea scale of grade 2–4 at the baseline (80.6%), which was reduced to 65.8% at 6 months and further reduced to 62.1% at 12 months, indicating that patients showed a significant improvement in clinically relevant dyspnea at 6 and 12 months compared to at the baseline (Table 6, Figure 4a).

Among patients without any comorbidities, 69.7% had an mMRC dyspnea scale of stage 2–4 at the baseline, reduced to 59.1% at 6 and 56.7% at 12 months (Table S9, Figure 4b). In patients with at least one comorbidity, however, 83.9% had an mMRC dyspnea scale of stage 2–4 at the baseline, while 67.8% and 63.9% of the patients had mMRC dyspnea scales of stage 2–4 at 6 and 12 months, respectively. These results indicate that patients with comorbidities had initially higher levels of dyspnea compared to patients without comorbidities, and both groups demonstrated an improvement in dyspnea at 6 and 12 months after treatment initiation (Table S10, Figure 4c).

In the group of patients with cardiovascular and/or metabolic diseases, 84.9% had mMRC dyspnea scales of stage 2–4 at the baseline, which was reduced to 68.7% and 64.5% at 6 and 12 months, respectively. These results indicate that these patients showed an improvement in dyspnea at 6 and 12 months compared to at the baseline, while their deteriorated health compared to the patients without any comorbidities had an effect on the severity of their dyspnea (Table S11, Figure 4d).

### 3.6. Satisfaction from the Elpenhaler^®^ Device as Assessed by the FSI-10 Questionnaire

The mean total score of the FSI-10 questionnaire was 42.8 (with a maximum total score of 50) and all the mean scores to the 10 questions were ≥4 (out of 5) points, suggesting that in general patients were satisfied by the usability of the Elpenhaler^®^ device. The question with the highest mean score for the total number of patients was the one regarding the ease of application of the inhaler to the lips (4.5 ± 0.6), while the one with the lowest mean score was associated with the ease of transportation of the inhaler (4.0 ± 0.9) (Table S12).

### 3.7. Safety Data

Information on patients with AEs is presented in Table S13. Overall, 12 patients (1.2%) had one AE throughout the study period, with 11 of them having a severe adverse event (SAE). Only one patient had a study treatment-related AE. Ten of the 11 SAEs led to death while 1 SAE required hospitalization. Six AEs were reported as deaths, while the remaining 4 deaths were attributed to cardiac arrest, cardiac failure and postoperative complication of inguinal hernia surgery and after thoracic surgery (Table S14).

## 4. Discussion

This is an open-label, 12-month observational prospective study with interesting data demonstrating the beneficial profile of fixed-dose SFC via the Elpenhaler^®^ device, when used in the routine care setting of Greece, examining changes in lung function and dyspnea at 6 and 12 months from the baseline with special focus on the presence of comorbidities. More specifically, a statistically significant improvement was observed in FEV1-predicted, FVC-predicted and mMRC scale values between consecutive visits, irrespective of the presence of comorbidities. Additionally, satisfaction from the Elpenhaler^®^ device, as assessed by the FSI-10 questionnaire at 6 months, was also recorded. Satisfactorily, 67.7% of the patients were vaccinated at least once, with 47.4% patients being vaccinated both for influenza and pneumococcus, which could be attributed to the close follow-up of expert respiratory physicians.

The beneficial effect of fixed-dose SFC therapy in patients with moderate to very severe COPD has been shown in previous studies, which demonstrated improvements in symptoms, reduced the number of exacerbations and improved lung function. Indeed, numerous publications support the use of SFC in COPD [16]. Our work is the first observational study of the SFC combination with the Elpenhaler^®^ device in patients with different stages of COPD severity in Greece, demonstrating that the aforementioned LABA/ICS combination significantly increased FEV1 at 6 and 12 months from the baseline, and the same applied for the FVC, FEV1/FVC ratio. The improvement in FEV_1_ from the baseline was more noticeable among patients without comorbidities, compared to patients with comorbidities, both at V1 and at V2. This effect can be both attributed to the efficacy of the medication and to the fact that patients were closely monitored at regular intervals (every 6 months) by experts. The improvement in FEV_1_ was observed regardless of the COPD severity at the baseline, being more prominent in GOLD groups B and C. Importantly, the majority of our study participants were symptomatic, with moderate to very severe airflow limitation and classified to GOLD groups C and D, despite the use of previous treatments, which may explain the beneficial effects of inhaled fluticasone in lung function of these patients. Additionally, the choice of the specific LABA/ICS combination was based on the clinical judgment of the participating experienced respiratory physicians.

In the univariate analysis, significant factors determining the change of FEV_1_ during the 12-month duration of the study were BMI, GOLD 2020 groups, mMRC dyspnea scale and the presence of comorbidities, a finding that remained unaffected in multiple linear regression analyses. These findings verify data from previous studies. A meta-analysis of 5 RCTs demonstrated the “obesity paradox” in patients with COPD; individuals with low BMI showed accelerated lung function deterioration, while obesity exhibited a protective effect over FEV_1_ annual reduction rate [17]. Moreover, in a cohort study of 72,683 COPD patients with a maximum follow-up of 13 years, a high mMRC dyspnea scale correlated to an accelerated rate of FEV1 and FVC decline [18]. The prevalence of comorbidities has been associated with the rapid decline of FEV_1_, an observation that could be attributed to the “spillover” of inflammatory mediators from the respiratory system into systemic circulation, thus heightening the impact of different comorbid conditions [19].

LABA/LAMA fixed-dose combinations are superior to LAMA or LABA/ICS in patients with stable moderate-to-very severe COPD, as a meta-analysis of 23 RCTs showed [20]. However, our aim was to evaluate the possible changes in lung function, in COPD patients with at least one comorbidity versus those without comorbidities, treated with a fixed-dose SFC in a real-world setting. Therefore, we could not exclude patients receiving LAMA. Moreover, our study population consisted of patients with moderate to very severe COPD, the majority of which receives by definition a LAMA in its regular treatment.

Additionally, improvement in dyspnea, which was noticeable 26 weeks after the study initiation, was shown. The functional impact of breathlessness in COPD can be assessed using the Medical Research Council dyspnea scale (MRC) or mMRC, which is more widely used [21,22,23]. The mMRC is a valid and reliable instrument for the assessment of dyspnea in COPD since it is easy to administer and interpret and can be completed by respondents independently in around 30 s [24]. Notably, the mMRC is an important independent predictor of 1- and 5-year mortality in COPD patients [25] with a study showing mMRC to be a better predictor of death than airway obstruction [26,27,28,29]. For this reason, GOLD 2020 recommends the use of the mMRC for categorizing COPD burden and guiding its management [2,30]. Furthermore, a domain-level analysis of Patient-Reported Outcomes (PRO) measures showed that breathlessness and its impact on patients were the most interpreted reported domains [31]. BDI and TDI, which are also frequently used instruments for assessing dyspnea, are interviewer-administered (except the self-administered computerized version) and not direct reports from patients. Consequently, there is a risk of potential interviewer bias during their completion [32]. The significant reduction in patients with clinically relevant dyspnea (i.e., in those with mMRC levels of 2 or higher) at 6 and 12 months in this study, both in patients with and without comorbidities or cardiovascular disease, further supports the clinical effectiveness of the SFC combination in real-life conditions.

The feeling of satisfaction through inhaler devices is a very crucial point in the management of chronic airway diseases, enhancing both adherence and disease control [33]. Until now, no inhaler meets the criteria of the ideal inhaler device, despite the fact that several types of devices are currently available [34,35]. There is strong evidence linking inhaler satisfaction with patient adherence and improved clinical outcomes, and that’s why the issue of patient satisfaction has been increasingly addressed over the last few years [36,37,38]. Patients who use their inhaler properly are more likely to feel more satisfied with their treatment, which is associated with better compliance and, therefore, complete control and better course of their disease [39]. The very recently translated and validated FSI-10 questionnaire is the only standardized questionnaire for use in Greece [40]. Response scales of other instruments used in inhaler satisfaction studies range from open-ended questions, through unclear response scales, to visual analog and Likert scales [41,42]. In fact, the Likert measuring scale used in the FSI-10 questionnaire has been proved to have better predictive performance compared with the visual analog scale [41]. In Greece, in real-life clinical practice, the FSI-10 questionnaire has been used to evaluate satisfaction both in patients with asthma and COPD [13]. The results from this study indicate that this specific questionnaire is understandable, easy to interpret, and has satisfactory measurement properties. It also showed a good association between questions and a positive contribution of the score of each question to the total score. Moreover, the questionnaire as a whole demonstrates very good internal consistency and no redundancies. However, no data are available regarding the sensitivity or the minimum clinically significant difference of the FSI-10, which accounts for the interim and exploratory nature of the interpretation of its results.

This is the first observational, multicenter study of the fixed-dose SFC with the Elpenhaler^®^ device in patients diagnosed with COPD, for whom the choice of ICS/LABA was based on the treating physician’s decision. Other real-world studies confirm the significance of our findings. In fact, a recent cohort study managed to replicate the COPD TORCH trial selection procedures and inclusion/exclusion criteria in real-world data and to obtain highly comparable relative effect estimates to the TORCH [43]. Nevertheless, in the same study, replication of placebo-controlled analyses was not possible. It is of note that the demographic characteristics of the patients included in the present study are similar to those of patients including in other studies prescribing the real-life use of fluticasone propionate/salmeterol in patients with COPD [44].

COPD is associated with multiple comorbidities [45], which contribute to poor health outcomes, high resource use and increased health-care costs [46]. Cardiovascular diseases (CVD) are common among COPD patients, while both diseases share common pathophysiological mechanisms and risk factors. The prevalence of CVD varies among patients with COPD between 13 and 68% [47]. A number of arguments can be used to explain our findings, namely, the significant improvement of lung function and mMRC dyspnea score at 6 and 12 months from the baseline, with fixed-dose SFC for patients with cardiovascular and/or metabolic comorbidities. Firstly, COPD patients with CVD seem to benefit from ICS, since CVD reflects an increased inflammatory state related to COPD [48], and under certain conditions, it may be a driver for exacerbations [49] and the severity of COPD [50]. Indeed, a subsequent analysis of cardiovascular-related mortality of the TORCH study found a positive effect of the FP/SAL combination in terms of CV-related outcomes [51], which further implies a potential beneficial impact of ICS use in COPD patients with CVD. Furthermore, results from the WISDOM trial have shown that when ICS withdrawal was completed, a statistically important between-group difference in FEV1 was observed in favor of ICS [52]. Based on these observations, we may hypothesize that ICS may be useful as add-on therapy also in COPD patients suffering from cardiovascular comorbidities. The prevalence of metabolic syndrome appears to be twice as high in COPD patients compared to the general population, and a number of studies have demonstrated this prevalence to be 21–62%. Impressively, almost 50% of COPD patients present with one or more components of the metabolic syndrome [53]. COPD patients with metabolic syndrome experience a more severe form of the disease reflected by more dyspnea, impaired lung function and the need to use more drugs (e.g., inhalation of glucocorticoids) to achieve disease control [54]. The improvement in breathlessness and lung function in patients with comorbidities further supports the potential beneficial effects of LABA/ICS combinations in patients with cardiovascular disease with an indication for these drugs.

The present study has certain limitations. First, the lack of data on exacerbations during the study due to the study design, the 6-month intervals in the follow-up visit and the absence of a clinical diary did not allow the appropriate collection of such events. Second, all patients were enrolled by respiratory physicians practicing in outpatient hospital clinics and in private offices, suggesting that the study results are generalizable to ambulatory patients under specialist care. However, the fact that patients were recruited from 71 different practices supports the generalizability of our findings. While the results indicated significant benefits in the entire study population, which underlines the wide applicability of the fixed-dose SFC combination, the extent of these benefits may in fact have been blurred due to other co-founding factors. Another limitation of our study is that since it was conducted between May 2017 and December 2017, when GOLD 2016 guidelines were in use, no data about the blood eosinophil count in the choice of corticosteroid inhalation therapy were collected. Finally, the absence of a comparator treatment arm limits the results of changes from the baseline. However, the fact that SFC combinations have been used for a long time in patients with COPD with comparable results in clinical trials further supports the observed improvements in dyspnea, while at the same time the spirometry measures are objective and reliable. Notably, the study had a very low attrition rate, thus preventing bias arising from selectively missing data from patients with early treatment discontinuation as a result of inadequate response.

## 5. Conclusions

The present study in 1016 Greek patients with moderate to very severe COPD confirms the previously reported efficacy and safety of the fixed-dose SFC in RCTs, supporting its effectiveness and tolerability in a real-world setting. Improvement in lung function parameters and a reduction of dyspnea scores on the mMRC scale in the total study population were observed irrespective of the presence of comorbidities and COPD severity at the baseline. These results altogether provide evidence of the effectiveness of the SFC combination in the Elpenhaler^®^ device in a wide range of COPD patients under real-world conditions.

## Figures and Tables

**Table 1:** Demographic and baseline characteristics of study participants.

Demographics	(Ν, %)
Age—years	N = 1016
Mean ± SD	69.5 ± 9.5
Gender—no. (%)	N = 1016
Female	264 (26)
BMI—kg/m^2^	N = 1016
Mean ± SD	28.6 ± 5.3
Years with COPD	N = 1016
Mean ± SD	7.3 ± 6.0
mMRC dyspnea scale—no. (%)	N = 1016
Stage 0	7 (0.7)
Stage 1	190 (18.7)
Stage 2	457 (45)
Stage 3	321 (31.6)
Stage 4	41 (4)
Classification according to GOLD spirometric stages—no. (%)	N = 1016
GOLD 1 (Mild)	0 (0)
GOLD 2 (Moderate)	471 (46.4)
GOLD 3 (Severe)	483 (47.5)
GOLD 4 (Extremely severe)	62 (6.1)
Classification according to GOLD 2020 Groups—no. (%)	N = 1016
Group A	56 (5.5)
Group B	286 (28.1)
Group C	326 (32.1)
Group D	348 (34.3)
Previous COPD treatment	(N, %)
No. (%) of patients with no past COPD treatment	199 (19.6)
No. (%) of patients with at least one past COPD treatment	817 (80.4)
Previous COPD maintenance treatment *—no. (%)	N = 817
LABA	66 (8.1)
LAMA	244 (29.8)
LABA + LAMA	322 (39.4)
Roflumilast	29 (3.5)
Theophylline	18 (2.2)
Other treatment **	174 (21.3)

* % of the total number of study patients, Ν = 1016; ** Other is SABA and/or SAMA, mucolytics etc.

**Table 2:** Comorbidities of participants in the study.

Comorbidities	(Ν, %)
No. (%) of patients with no comorbidities	238 (23.4)
No. (%) of patients with at least one comorbidity	778 (76.6)
Number of comorbidities per patient—no. (%)	
1	256 (32.9)
2	233 (29.9)
>2	289 (37.2)
Number of patients per comorbidity category—no. (%) *	N = 778
Cardiovascular diseases	653 (83.9)
Coronary artery disease	175 (22.5)
Arterial hypertension	557 (71.6)
Cardiac failure	122 (15.7)
Peripheral arterial disease	41 (5.3)
Pulmonary embolism	9 (1.2)
Other	88 (11.3)
Metabolic diseases	373 (47.9)
Diabetes Mellitus Type ΙΙ	169 (21.7)
Dyslipidemia	265 (34.1)
Other	33 (4.2)
Psychiatric diseases	160 (20.6)
Depression	83 (10.7)
Anxiety disorder	88 (11.3)
Other	11 (1.4)
Malignancies	56 (7.2)
Lung	13 (1.7)
Lymphoma	0 (0)
Breast	9 (1.2)
Other	32 (4.1)
Other diseases	132 (17)
Osteoporosis	47 (6)
Other	84 (10.8)
No. (%) of patients with cardiovascular and metabolic diseases	711 (91.4)

* % of the total number of patients with comorbidities, Ν = 778.

**Table 3:** Spirometry results at 0, 6 and 12 months and change from baseline and 6 months for the total number of patients.

Spirometry	Baseline (Day 0) N = 1016	Visit 1 (6 Months) N = 792	Visit 2 (12 Months) N = 746
FEV_1_ predicted L—			
Mean ± SD	1.35 ± 0.37	1.50 ± 0.42	1.56 ± 0.45
FEV_1_% predicted—%			
Mean ± SD	48.43 ± 8.61	54.17 ± 10.87	56.47 ± 11.82
Change of FEV_1_ (L) from baseline (Mean ± SD, *p*-value)	-	0.15 ± 0.22, <0.0001	0.21 ± 0.25, <0.0001
Change of FEV_1_ (L) between 6 and 12 months * (Mean ± SD, *p*-value)	-	-	0.06 ± 0.19, <0.0001
FVC predicted L—			
Mean ± SD	2.48 ± 0.72	2.59 ± 0.72	2.67 ± 0.76
FVC % predicted			
Mean ± SD	68.61 ± 14.42	71.61 ± 14.07	74.19 ± 15.42
Change of FVC (L) from baseline (Mean ± SD, *p*-value)	-	0.11 ± 0.35, <0.0001	0.19 ± 0.41, <0.0001
Change of FVC (L) between 6 and 12 months * (Mean ± SD, *p*-value)	-	-	0.09 ± 0.38, <0.0001
FEV_1_/FVC ratio			
Mean ± SD	0.58 ± 0.08	0.62 ± 0.10	0.62 ± 0.11
Change of FEV_1_/FVC from baseline (Mean ± SD, *p*-value)	-	0.036 ± 0.07, <0.0001	0.039 ± 0.07, <0.0001
Change of FEV_1_/FVC between 6 and 12 months * (Mean ± SD, *p*-value)	-	-	0.002 ± 0.08, 0.595

* Changes between 6 and 12 months have been assessed by ANCOVA using baseline spirometry values as covariates.

**Table 4:** Change of FEV_1_ (L) between 0–6, 0–12 and 6–12 months based on GOLD COPD 2020 groups.

Spirometry	ΔFEV1 0–6 Months (Mean ± SD)	ΔFEV1 0–12 Months (Mean ± SD)	ΔFEV1 6–12 Months (Mean ± SD)
GOLD 2020 Groups			
(* *p*-value)	0.015	<0.001	0.014
Group A	0.07 ± 0.23	0.19 ± 0.25	0.12 ± 0.18
Group B	0.17 ± 0.19	0.25 ± 0.24	0.07 ± 0.15
Group C	0.19 ± 0.22	0.26 ± 0.27	0.07 ± 0.23
Group D	0.13 ± 0.20	0.17 ± 0.23	0.03 ± 0.18

* Changes between 0–6, 0–12 and 6–12 months have been assessed by Kruskal–Wallis test.

**Table 5:** Univariate and multiple linear regression analysis of FEV_1_ change between baseline visit and 12 months after (V2).

Variables	Univariate			Multivariate		
	*β*	95% CI	* *p*	*β*	95% CI	* *p*
Age	−0.030	−0.003, 0.001	0.418	0.16	−0.002, 0.002	0.689
Gender (female)	−0.026	−0.055, 0.026	0.481	−0.016	−0.050, 0.032	0.656
BMI	0.085	0.001, 0.007	0.020	0.083	0.001, 0.007	0.024
mMRC	−0.087	−0.051, −0.005	0.018	−0.072	−0.050, 0.000	0.047
Comorbidities	−0.089	−0.098, −0.010	0.015	−0.078	−0.092, −0.003	0.035
GOLD 2020 Groups	−0.093	−0.046, −0.006	0.011	−0.099	−0.050, −0.006	0.013
Baseline FEV_1_	0.018	−0.037, 0.061	0.627	−0.016	−0.063, 0.041	0.682

* Multiple linear regression analysis was used.

**Table 6:** mMRC dyspnea scale at 0, 6 and 12 months and difference between study visits for the total number of patients.

mMRC Dyspnea Scale	Baseline (Day 0) N = 1016, %	Visit 1 (6 Months) N = 992, %	Visit 2 (12 Months) N = 966, %
Stage 0	7 (0.7)	24 (2.4)	67 (6.9)
Stage 1	190 (18.7)	315 (31.8)	299 (31)
Stage 2	457 (45)	466 (47)	434 (44.9)
Stage 3	321 (31.6)	172 (17.3)	148 (15.3)
Stage 4	41 (4)	15 (1.5)	18 (1.9)
Difference in mMRC scale between visits * (*p*-value)	<0.0001		

* Kruskal–Wallis test was used.

## Data Availability

All data generated or analyzed during this study are included in this published article. Anonymized data will be shared by request from any qualified investigator.

## Supplementary Materials

The following are available online at https://www.mdpi.com/article/10.3390/jpm11111159/s1, Table S1: Smoking status at baseline and during the study, Table S2: Vaccination during the study, Table S3: COPD treatment during the study, Table S4: Spirometry results at 0, 6 and 12 months and change from baseline and 6 months for the patients without any comorbidities, Table S5: Spirometry results at 0, 6 and 12 months and change from baseline and 6 months for the patients with at least one comorbidity, Table S6: Spirometry results at 0, 6 and 12 months and change from baseline and 6 months for the patients with cardiovascular and/or metabolic diseases, Table S7: Change of FEV_1_ (L) between 0–6, 0–12 and 6–12 months based on GOLD spirometric stages, Table S8: Change of FEV_1_ (L) between 0–6, 0–12 and 6–12 months based on mMRC dyspnea scale, Table S9: mMRC dyspnea scale at 0, 6 and 12 months and difference between study visits for the patients without any comorbidities, Table S10: mMRC dyspnea scale at 0, 6 and 12 months and difference between study visits for the patients with at least one comorbidity, Table S11: mMRC dyspnea scale at 0, 6 and 12 months and difference between study visits for the patients with cardiovascular and/or metabolic diseases, Table S12: Satisfaction from the device, as assessed by FSI-10 questionnaire at 6 months for the total number of patients, Table S13: Summary characteristics of the recorded Adverse Events and Table S14: List of Adverse Events (serious and non-serious) by SOC and PT.

## Abbreviations

AE: adverse event; BMI: body mass index; COPD: Chronic Obstructive Pulmonary Disease; FEV_1_: forced expiratory volume at 1 second; FSI-10: Feeling of Satisfaction with the Inhaler; FVC: forced vital capacity; ICS: inhaled corticosteroids; LABA: long-acting beta-agonist; LAMA: long-acting muscarinic antagonists; mMRC: modified Medical Research Council; RCTs: randomized controlled trials; SAE: serious adverse event; SFC: salmeterol and fluticasone propionate combination.
