# Supplementary material for: Evaluation of the Clinical Effectiveness of the Salmeterol/Fluticasone Fixed-Dose Combination Delivered via the Elpenhaler® Device in Greek Patients with Chronic Obstructive Pulmonary Disease and Comorbidities: The AEOLOS Study
Source: J Pers Med. 2021 Nov 8;11(11):1159. doi: 10.3390/jpm11111159 (PMC8621702; doi:10.3390/jpm11111159)
Supplement: Supplementary file 1 [file jpm-11-01159-s001.zip › jpm-1440673-supplementary.pdf]

Evaluation of the clinical effectivenessof the salmeterol/fluticasone fixed dose combination delivered via the Elpenhaler® device in Greek patients with Chronic Obstructive Pulmonary Diseaseand comorbidities: the AEOLOS study

Supplementary appendix

Table S1: Smoking status at baseline and during the study

**Smoking status**	***(N, %)***
**Smoking status– no. (%)**	***N=1016***
Non-smoker	68 (6.7)
Ex smoker	536 (52.8)
Time from discontinuation – years	
Mean ± SD	10.4±8.4
Pack-years	
Mean ± SD	59.3±29.0
Current smoker	412 (40.5)
Years of smoking *(Mean ± SD)*	44.7±11.4
Pack-years	
Mean ± SD	60.4±28.4
No. (%) of patients who discontinued smoking up to 6 months after study initiation	111 (10.9)
No. (%) of patients who discontinued smoking between 6-12 months after study initiation	66 (6.5)

Table S2: Vaccination during the study

**Vaccination**	***(N, %)***
**No. (%) of patients with at least one vaccination**	688 (67.7)
Influenza	632 (62.2)
Pneumonococcus	530 (52.2)
Both	482 (47.4)
Other	6 (0.9)

*% of the total number of study patients, N=1016

Table S3: COPD treatment during the study

COPD treatment	*(N, %)*
**No. (%) of patients receiving SFC Elpenhaler®at study initiation**	1016
**Monotherapy with SFC Elpenhaler®at baseline–** ***no. (%)***	421 (41.4)
**Combination therapy with SFC Elpenhaler®at baseline* –** ***no. (%)***	595 (58.6)
ICS+LABA+LAMA	486 (47.8)
ICS+LABA+Romiluflast	14 (1.4)
ICS+LABA+LAMA+Romiluflast	27 (2.7)
ICS+LABA+LAMA+Theophylline	45 (4.5)
ICS+LABA+SABA	9 (0.9)
ICS+LABA+LAMA+SABA	9 (0.9)
Other	5 (0.5)
**Change in COPD treatment at 6 months* –** ***no. (%)***	26 (2.6)
Addition of LAMA	14 (1.4)
Addition of LABA+LAMA	4 (0.4)
Addition of ICS	2 (0.2)
Addition of Romiluflast	2 (0.2)
Addition of SABA	1 (0.1)
LAMA discontinuation, monotherapy with SFC Elpenhaler®	2 (0.2)
LAMA replaced	1 (0.1)
**Change in COPD treatment at 12 months* –** ***no. (%)***	7 (0.7)
Addition of LABA+LAMA	2 (0.2)
Addition of Roflumilast	1 (0.1)
Addition of ICS	1 (0.1)
LAMA discontinuation, monotherapy with SFC Elpenhaler®	1 (0.1)
Addition of SABA+SAMA	1 (0.1)
Holistic diet	1 (0.1)

*% of the total number of study patients, N=1016

Table S4: Spirometry results at 0, 6 and 12 months and change from baseline and 6 months for the patients without any comorbidities

**Spirometry**	***Patients without comorbidities (N=238)***		
	Baseline (Day 0) N=238	***Visit 1 (6 months) N=158***	***Visit 2 (12 months) N=158***
FEV_1 predicted Lit. – Mean ± SD	1.48 ± 0.40	1.67 ± 0.48	1.76 ± 0.52
FEV_1% predicted – % Mean ± SD	49.90 ± 8.23	55.95 ±10.96	59.16 ± 11.84
**Change of FEV_1 (L) from baseline** *(Mean* ± SD, *p-value)*	-	0.19 ± 0.24, <0.0001	0.28 ± 0.27, <0.0001
**Change of FEV_1 (L) between 6 and 12 months*** *(Mean* ± SD, *p-value)*	-	-	0.08 ± 0.24, <0.0001
FVC predicted L- Mean ± SD	2.78 ± 0.74	2.88 ± 0.74	2.99 ± 0.76
FVC % predicted Mean ± SD	72.25 ± 13.81	74.51 ± 13.67	77.77 ± 13.26
**Change of FVC (L) from baseline** *(Mean* ± SD, *p-value)*	-	0.10 ± 0.37, 0.002	0.21 ± 0.37, <0.0001
**Change of FVC (L) between 6 and 12 months*** *(Mean* ± SD, *p-value)*	-	-	0.11 ± 0.31, 0.004
FEV_1/FVC ratio Mean ± SD	0.58 ± 0.08	0.63 ± 0.09	0.62± 0.10
**Change of FEV_1/FVC from baseline** *(Mean* ± SD, *p-value)*	-	0.05 ± 0.07,<0.0001	0.04 ± 0.07,<0.0001
**Change of FEV_1/FVC between 6 and 12 months*** *(Mean*± SD, *p-value)*	-	-	-0.003 ± 0.06, 0.534

*Changes between 6-12 months have been assessed by ANCOVA, using baseline spirometry values as covariates

Table S5: Spirometry results at 0, 6 and 12 months and change from baseline and 6 months for the patients with at least one comorbidity

Spirometry	*Patients with at least one comorbidity (N=778)*		
	Baseline (Day 0) N=778	*Visit 1 (6 months) N=634*	*Visit 2 (12 months) N=588*
FEV_1 predicted L – Mean ± SD	1.31 ± 0.35	1.46 ± 0.39	1.51 ± 0.41
FEV_1% predicted – % Mean ± SD	48.10 ± 8.69	53.70 ± 10.79	55.75 ± 11.73
**Change of FEV_1 (L) from baseline** *(Mean ± SD, p-value)*	-	0.15 ± 0.21, <0.0001	0.20 ± 0.24, <0.0001
**Change of FEV_1 (L) between 6 and 12 months*** *(Mean ± SD, p-value)*	-	-	0.05 ± 0.18, <0.0001
FVC predicted L- Mean ± SD	2.39 ± 0.69	2.50 ± 0.69	2.59 ± 0.73
FVC % predicted Mean ± SD	67.21 ± 14.39	70.55 ± 14.08	73.10 ± 15.82
**Change of FVC (L) from baseline** *(Mean ± SD, p-value)*	-	0.11 ± 0.34, <0.0001	0.20 ± 0.42, <0.0001
**Change of FVC (L) between 6 and 12 months*** *(Mean ± SD, p-value)*	-	-	0.09 ± 0.40, <0.0001
FEV_1/FVC ratio Mean ± SD	0.58 ± 0.08	0.61 ± 0.10	0.62 ± 0.12
**Change of FEV_1/FVC from baseline** *(Mean ± SD, p-value)*	-	0.036 ± 0.08, <0.0001	0.041 ± 0.10, <0.0001
**Change of FEV_1/FVC between 6 and 12 months*** *(Mean ± SD, p-value)*	-	-	0.002 ± 0.09, 0.555

*Changes between 6-12 months have been assessed by ANCOVA, using baseline spirometry values as covariates

Table S6: Spirometry results at 0, 6 and 12 months and change from baseline and 6 months for the patients with cardiovascular and/or metabolic diseases

**Spirometry**	***Patients with cardiovascular and/or metabolic diseases N=711)***		
	Baseline (Day 0) N=714	***Visit 1 (6 months) N=573***	***Visit 2 (12 months) N=536***
FEV_1 predicted L – Mean ± SD	1.30 ± 0.34	1.44 ± 0.38	1.50 ± 0.40
FEV_1% predicted – % Mean ± SD	47.98 ± 8.76	53.61 ± 10.85	55.68 ± 11.65
**Change of FEV_1 (L) from baseline** *(Mean± SD, p-value)*	-	0.14 ± 0.21, <0.0001	0.20 ± 0.24, <0.0001
**Change of FEV_1 (L) between 6 and 12 months*** *(Mean ± SD, p-value)*	-	-	0.06 ± 0.18, <0.0001
FVC predicted L- Mean ± SD	2.39 ± 0.68	2.50 ± 0.67	2.58 ± 0.71
FVC % predicted Mean ± SD	66.97 ± 14.41	70.59 ± 13.97	73.18 ± 15.84
**Change of FVC (L) from baseline** *(Mean ± SD, p-value)*	-	0.11 ± 0.34, <0.0001	0.20 ± 0.42, <0.0001
**Change of FVC (L) between 6 and 12 months*** *(Mean ± SD, p-value)*	-	-	0.09 ± 0.41, <0.0001
FEV_1/FVC ratio Mean ± SD	0.59 ± 0.08	0.61 ± 0.10	0.62 ± 0.12
**Change of FEV_1/FVC from baseline** *(Mean ± SD, p-value)*	-	0.035 ± 0.08, <0.0001	0.038 ± 0.10,<0.0001
**Change of FEV_1/FVC between 6 and 12 months*** *(Mean ± SD, p-value)*	-	-	0.0007 ± 0.08, 0.865

*Changes between 6-12 months have been assessed by ANCOVA, using baseline spirometry values as covariates

Table S7: Change of FEV_1 (L) between 0-6, 0-12 and 6-12 months based on GOLD spirometric stages

**Spirometry**	***Δ FEV1 0-6 months*** *(Mean* ± SD*)*	***Δ FEV1 0-12 months*** *(Mean* ± SD*)*	***Δ FEV1 6-12 months*** *(Mean* ± SD*)*
**GOLD spirometric stages**			
(p-value)	0.022	0.087	0.362
GOLD 1	-	-	-
GOLD 2	0.14 ± 0.19	0.20 ± 0.24	0.06 ± 0.19
GOLD 3	0.18 ± 0.22	0.23 ± 0.26	0.05 ± 0.19
GOLD 4	0.13 ± 0.22	0.15 ± 0.20	0.02 ± 0.17

*Changes between 0-6, 0-12 and 6-12 months have been assessed byKruskal–Wallis test

Table S8: Change of FEV_1 (L) between 0-6, 0-12 and 6-12 months based on mMRCdyspnea scale

**Spirometry**	***Δ FEV1 0-6 months*** *(Mean* ± SD*)*	***Δ FEV1 0-12 months*** *(Mean* ± SD*)*	***Δ FEV1 6-12 months*** *(Mean* ± SD*)*
**mMRC dyspnea scale**			
(p-value)	0.200	0.083	0.060
**Stage 0**	0.08 ± 0.15	0.23 ± 0.37	0.16 ± 0.16
**Stage 1**	0.17 ± 0.20	0.26 ± 0.25	0.09 ± 0.17
**Stage 2**	0.17 ± 0.22	0.23 ± 0.26	0.06 ± 0.21
**Stage 3**	0.14 ± 0.21	0.18 ± 0.24	0.04 ± 0.19
**Stage 4**	0.14 ± 0.14	0.15 ± 0.17	0.13 ± 0.12

*Changes between 0-6, 0-12 and 6-12 months have been assessed byKruskal–Wallis test

Table S9: mMRC dyspnea scale at 0, 6 and 12 months and difference between study visits for the patients without any comorbidities

mMRC dyspnea scale	*Patients without comorbidities (N=238)*		
	Baseline (Day 0) N=238, %	***Visit 1 (6 months) N=235, %***	***Visit 2 (12 months) N=231, %***
**Stage 0**	5 (2.1)	8 (3.4)	32 (13.9)
**Stage 1**	67 (28.2)	88 (37.4)	68 (29.4)
**Stage 2**	104 (43.7)	110 (46.8)	97 (42)
**Stage 3**	60 (25.2)	28 (11.9)	30 (13)
**Stage 4**	2 (0.8)	1 (0.4)	4 (1.7)
**Difference in mMRC scale between visits****(p-value)*	<0.0001		

* Kruskal-Wallis test was used

Table S10: mMRC dyspnea scale at 0, 6 and 12 months and difference between study visits for the patients with at least one comorbidity

mMRC dyspnea scale	*Patients with at least one comorbidity (N=778)*		
	Baseline (Day 0) N=778, %	***Visit 1 (6 months) N=757, %***	***Visit 2 (12 months) N=735, %***
**Stage 0**	2 (0.3)	16 (2.1)	35 (4.8)
**Stage 1**	123 (15.8)	227 (30)	231 (31.4)
**Stage 2**	353 (45.4)	356 (47)	337 (45.9)
**Stage 3**	261 (33.5)	144 (19)	118 (16.1)
**Stage 4**	39 (5)	14 (1.8)	14 (1.9)
**Difference in mMRC scale between visits****(p-value)*	<0.0001		

* Kruskal-Wallis test was used

Table S11: mMRC dyspnea scale at 0, 6 and 12 months and difference between study visits for the patients with cardiovascular and/or metabolic diseases

mMRC dyspnea scale	*Patients with cardiovascular and/or metabolic diseases (N=711)*		
	Baseline (Day 0) N=714, %	***Visit 1 (6 months) N=693, %***	***Visit 2 (12 months) N=671, %***
**Stage 0**	2 (0.3)	14 (2)	26 (3.9)
**Stage 1**	106 (14.8)	203 (29.3)	212 (31.6)
**Stage 2**	322 (45.1)	327 (47.2)	306 (45.6)
**Stage 3**	246 (34.5)	136 (19.6)	114 (17)
**Stage 4**	38 (5.3)	13 (1.9)	13 (1.9)
**Difference in mMRC scale between visits****(p-value)*	<0.0001		

* Kruskal-Wallis test was used

Table S12: Satisfaction from the device, as assessed by FSI-10 questionnaire at 6 months for the total number of patients

**FSI-10 Questionnaire** *(Mean ±SD, Median)*	***Visit 1*** ***(6 months)*** ***N=993***
1. Has it been easy to learn how to use the inhaler?	4.23±0.75, 4.00
2. Was it easy to prepare the inhaler for use?	4.10±0.82, 4.00
3. Was it easy to use the inhaler?	4.27±0.74, 4.00
4. Was it easy to keep the inhaler clean and in good working condition?	4.38±0.67, 4.00
5. Was it easy to continue normal activities with the use of the inhaler?	4.37±0.69, 4.00
6. Did the inhaler fit your lips comfortably?	4.46±0.63, 5.00
7. Was using the inhaler easy in terms of size and weight?	4.21±0.74, 4.00
8. Was it easy to carry the inhaler with you?	4.00±0.91, 4.00
9. After you've used the inhaler, do you have the feeling that you used it correctly?	4.43±0.63, 5.00
10. Overall, considering your responses to the previous questions, were you satisfied with the inhaler?	4.34±0.62, 4.00
**Total FSI-10 score**	
Mean ±SD	42.80±5.48
Min, Max	13.00, 50.00

FSI: Feeling of Satisfaction with the Inhaler

Table S13: Summary characteristics of the recorded Adverse Events

**Adverse Events**	***(N, %)***
**No. (%) of patients studied**	1016
No. (%) of patients with at least one **AE**	12 (1.2)
No. (%) of patients with at least one **SAE**	11 (1.1)
No. (%) of patients with at least one**treatment-related AE**	1 (0.1)
**AE related to study drug –** ***no. (%)***	***N=12***
Yes	1 (8.3)
No	11 (91.7)
**Serious AE –** ***no. (%)***	***N=12***
Yes	11 (91.7)
No	1 (8.3)
**Seriousness criteria –** ***no. (%)***	***N=11***
Fatal	10 (90.9)
Required hospitalization	1 (9.1)

AE: adverse event; SAE: severe adverse event

Table S14: List of Adverse Events (serious and non-serious), by SOC and PT

System Organ Class (SOC)	Preferred Term (PT)	Total no. of AEs N=1016 (%[†])
Cardiacdisorders	Cardiac arrest	1 (0.1)
	Cardiac failure	1 (0.1)
General disorders and administration site conditions	Death	6 (0.6)
Infections	Oropharyngealgonococcalinfection	1 (0.1)
	Pneumonia*	1 (0.1)
Respiratory, thoracic and mediastinal disorders	Hypercapnia*	1 (0.1)
	Pickwickian syndrome*	1 (0.1)
Surgical and medical procedures	Inguinal hernia repair	1 (0.1)
	Thoracic operation	1 (0.1)

* One patient had 3 events: Pneumonia, hypercapnia, and pickwickian syndrome; [†]% of the total number of study patients
